# Injectable Cell-Laden Nanofibrous Matrix for Treating Annulus Fibrosus Defects in Porcine Model: An Organ Culture Study

**DOI:** 10.3390/life12111866

**Published:** 2022-11-12

**Authors:** Evan Roebke, Diego Jacho, Oliver Eby, Sulaiman Aldoohan, Haitham Elsamaloty, Eda Yildirim-Ayan

**Affiliations:** 1 Department of Bioengineering, College of Engineering, University of Toledo, Toledo, OH 43606, USA; 2 Department of Radiology, College of Medicine and Life Sciences, University of Toledo, Toledo, OH 43606, USA

**Keywords:** annulus fibrosus, tissue regeneration, ex vivo, organ culturing, porcine model, polycaprolactone, collagen type I, injectable, intervertebral disc, defect, biopsy punch, aggrecan, scleraxis, tenascin, anti-inflammatory genes

## Abstract

Lower back pain commonly arises from intervertebral disc (IVD) failure, often caused by deteriorating annulus fibrosus (AF) and/or nucleus pulposus (NP) tissue. High socioeconomic cost, quality of life issues, and unsatisfactory surgical options motivate the rapid development of non-invasive, regenerative repair strategies for lower back pain. This study aims to evaluate the AF regenerative capacity of injectable matrix repair strategy in ex vivo porcine organ culturing using collagen type-I and polycaprolactone nanofibers (PNCOL) with encapsulated fibroblast cells. Upon 14 days organ culturing, the porcine IVDs were assessed using gross optical imaging, magnetic resonance imaging (MRI), histological analysis, and Reverse Transcriptase quantitative PCR (RT-qPCR) to determine the regenerative capabilities of the PNCOL matrix at the AF injury. PNCOL-treated AF defects demonstrated a full recovery with increased gene expressions of AF extracellular matrix markers, including Collagen-I, Aggrecan, Scleraxis, and Tenascin, along with anti-inflammatory markers such as CD206 and IL10. The PNCOL treatment effectively regenerates the AF tissue at the injury site contributing to decreased herniation risk and improved surgical outcomes, thus providing effective non-invasive strategies for treating IVD injuries.

## 1. Introduction

Lower back pain (LBP) is one of the leading causes of disability in the United States. Nearly 80% of the population is affected by LBP at some point in life, with intervertebral disc (IVD) herniation causing approximately 300,000 spine-related surgeries annually in the United States [1,2,3,4]. While there are various causes of IVD herniation, all lead to the degeneration of the annulus fibrosus (AF), which eventually breaks down enough to allow the nucleus pulposus to herniate. To combat back pain, patients typically go through conservative approaches of physical therapy, spinal injections, or pain medication [5,6,7]. However, these procedures have shown limited patient improvement and have only palliative rather than actual regenerative effects [8,9]. If these conservative attempts have failed, a partial or microdiscectomy is a common surgical procedure for disc herniation [2,7,10,11], where the herniated material is removed, and the nerve is decompressed [7,12]. While surgical interventions are the current standard of care for intervertebral disc (IVD) complications, this treatment addresses only the symptoms but does not repair AF defects nor address the underlying cause of pathological IVDs.

The intervertebral disc is one of the most important organs in the human body, as it is responsible for bearing loads and enabling flexibility in the spine [13,14,15]. IVDs are comprised of three main structures: the superior and inferior vertebral endplates, the nucleus pulposus (NP), and the annulus fibrosus (AF) [3,16,17,18]. The endplates provide IVD nutrients through oxygen diffusion, while NP acts as a shock absorber for compressive forces acting on IVDs [6,13]. The AF comprises a highly ordered, angle-ply lamellar structure surrounding the NP and preventing herniation from gel-like NP tissue [19]. AF tissue comprises comparatively few cells that secrete ECM matrix components such as proteoglycans, glycoproteins, and elastin fibers [19], which give AF tissue the unique mechanical properties of viscoelasticity, anisotropy and nonlinearity [11]. Although physically strong, the AF has limited healing capacity [17,18] due to its avascularity and low cellularity. Thus, there is a great demand for cellular- and tissue-scaffold-based AF repair strategies to repair and regenerate AF tissues. 

Toward this end, early strategies of IVD regeneration seek to seal the damaged AF using synthetic and natural polymers [20,21]. While these strategies may halt disc herniation, they were not designed to regenerate the damaged AF tissue. Thus, a second-generation strategy has been immersed, focusing on designing biomimetic AF scaffolds with cells to mimic the AF’s fibrous structure and promote extracellular matrix repair. There are numerous prominent in vitro studies that successfully mimicked the angle-ply lamellae structure of AF using synthetic and natural polymers and demonstrated that seeded cells on these scaffolds proliferated and deposited collagen-rich extracellular matrix for repair [22,23,24,25]. The commonly used biomaterials in promising in vitro studies are collagen type-I and polycaprolactone (PCL). Collagen type-I is the primary ECM protein in most musculoskeletal tissues, including AF [26,27,28]. Its biomimetic capabilities are effective in therapeutic applications [3,5]. Yet, collagen type I-based AF tissue scaffold mechanical properties are unsuitable for the high magnitude of forces that AF tissue experiences during diurnal activities [29,30]. Thus, in general, for AF tissue regeneration, collagen type-I is admixed with synthetic polymers, including PCL. The PCL is an FDA-approved, biocompatible, and cost-effective biodegradable material used in IVD regeneration studies [7,18,31]. Thus, numerous prominent in vitro studies investigated the biphasic collagen-PCL scaffolds for AF regeneration [7,32,33,34]. 

As stated, numerous comprehensive biomimetic scaffold designs and material systems have been investigated using in vitro platforms for AF regeneration. Yet, it is unclear whether the success of in vitro studies can be translated into ex vivo IVD organ culturing and in vivo conditions. In this respect, the in vitro platforms are cost-effective and convenient, but in vitro studies do not comprehensively simulate the inherent complexity of the IVD with chemical, mechanical, nutritional, and metabolic factors sustaining the biological and mechanical functions of IVDs. In the last decade, there has been a great interest in utilizing ex vivo organ culture platforms for human or animal IVD culturing to study the injury, degeneration, and repair of IVDs [29,35]. Among the validated ex vivo models, the bovine caudal disc culturing has been adapted for many IVD regeneration studies [27,36,37] for providing a cost-effective option for mechanistic research. However, the bovine discs are too small compared to human IVDs and do not replicate the intradiscal pressure, biomechanical stress, and cell physiology of human IVDs. The large animal ex vivo models, including ovine [38,39] and porcine IVD culturing [2,40] are more relevant for potential clinically relevant tissue regenerative interventions for IVD repair. 

To this end, our objective was to investigate the regenerative capabilities of injectable yet nanofibrous collagen type-I and PCL scaffold (PNCOL) with encapsulated cells for annulus fibrosus repair under ex vivo porcine IVD organ culturing conditions. We have investigated the PNCOL-induced various musculoskeletal tissue regeneration, including bone, tendon, and annuls fibrosus in vitro [34,41,42], but its potential needed to be further assessed under more relevant in vivo conditions. Thus, we have utilized our validated IVD organ culture platform [43] to investigate the role of cell-laden PNCOL treatment on porcine annulus fibrosus defect regeneration under 14-day ex vivo organ culture. A comprehensive structural, molecular, and biological characterization of 14-day ex vivo IVD organ culturing demonstrated that cell-laden PNCOL injected into the annulus fibrous defect was effective for fully repairing the defect site with a newly regenerated matrix like the native AF ECM.

## 2. Materials and Methods

### 2.1. Porcine Intervertebral Disc Isolation 

Five cervical IVDs were harvested from skeletally mature female pigs immediately after sacrificing the animals. The soft tissues and fascia surrounding the vertebra were removed, and then the spinous and transverse processes were cut through the pedicles bilaterally. Using a commercial bandsaw, the cervical spine was cut through the vertebral bodies anteriorly and posteriorly. For each IVD, the cut was done 3 mm away from the endplates to preserve them. Thus, each sample has an anterior 3 mm vertebral bony part, an anterior intact endplate, an IVD, a posterior intact endplate, and a 3 mm posterior vertebral bony part. Following the isolation, IVDs were washed with 70% EtOH for 10 s and soaked in a sterile washing solution with phosphate-buffered saline (PBS), 3% penicillin/streptomycin, and 1.5% fungizone for 15 min. All processes were conducted under sterile conditions. The IVDs were then transferred to the X-ray facility for defect creation. 

### 2.2. X-ray Guided Intervertebral Disc Defect Creation 

**Figure 1:**
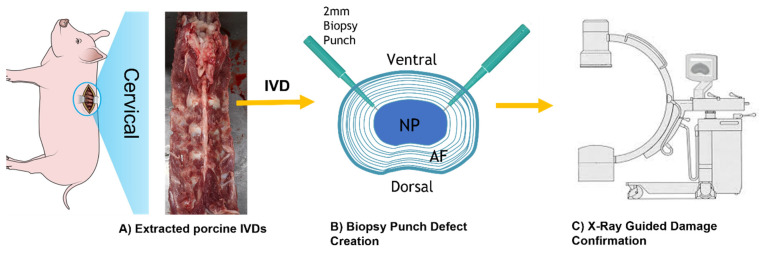
(**A**) Porcine spinal column extracted from a 6-month-old pig immediately after sacrificing the animal. The cervical section was isolated, and five cervical IVDs were used in each experiment. (**B**) The schematic representation of biopsy punch defect creations (not scaled) (**C**) The schematic of the X-ray C-Arm system used for IVD damage creation confirmation.

Within an hour after isolation, an annulus fibrosus defect was created on the right and left posterolateral side of the IVD while maintaining the NP intact using a 2 mm biopsy punch (Robbins Instruments Chatham, NJ, USA) following established protocols [44,45]. All IVDs were punched with a 2 mm biopsy punch, including the control samples. The 2 mm biopsy punch damage was selected to induce injuries of 25% of the IVD height. This demonstrates that puncture injuries of 20%-disc size or higher were required to create significant damage to the IVDs [46,47]. The course of the biopsy punch through the IVD was monitored using GE OEC 9000 mobile C-arm X-ray system (GE OEC Medical Systems, Salt Lake City, UT, USA) to confirm AF penetration without damaging endplates or NP. The X-ray system was operated under the fluoroscopy setting with the pulse generator at 73 kV & 2.4 mA/mAs. Automatic adjustment for image contrast and brightness was enabled. Figure 1 illustrates the steps in IVD isolation and X-ray-guided defect area creation. 

### 2.3. Synthesis of Cell-Laden Injectable Nanofibrous Scaffold and Implementation to the Defect Site

**Figure 2:**
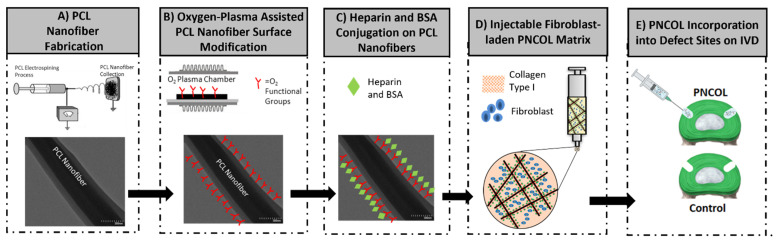
Fibroblast-laden PNCOL matrix synthesis for defective AF regeneration.

The injectable yet nanofibrous fibroblast-laden scaffold, called PNCOL, was synthesized based on our well-established protocol [34] through interspersing polycaprolactone (PCL) electrospun nanofibers within the fibroblast encapsulated neutralized collagen type-I solution. Figure 2 demonstrates the schematic representation of the injectable fibroblast-laden PNCOL matrix synthesis process.

Briefly, PCL (MW 45,000, Sigma-Aldrich, St. Louis, MI, USA) pellets were dissolved overnight in a 3:1 mixture of chloroform and methanol solution under the chemical hood at 16% *w*/*v*. The next day, the PCL solution was loaded into a syringe pump and extruded with a flow rate of 8 mL/h through a 20-gauge needle. A 20 kV potential was applied between the needle tip and a collector plate where PCL nanofibers were collected. Following 2 h of PCL nanofiber collection, the PCL fibers were transferred under the chemical hood for residual solvent evaporation overnight. The next day, the PCL nanofibers were homogenized using a high-speed homogenizer (Ultra-Turrax, IKA Works, Inc., Staufen, Germany) and functionalized using oxygen-plasma treatment for 3 min to reduce its hydrophobicity based on our prior studies [34]. The plasma-treated PCL nanofibers were then incubated with heparin (20 μg/mL) (Fisher Scientific, Hampton, NH, USA) and Bovine Serum Albumin (BSA) (1 mg/mL) with 40:2000 ratio at 37 °C and over 95% humidity for 1 h. Upon incubation, the PCL nanofibers were mixed within the neutralized collagen type-I solution with a 3% (*w*/*v*) concentration. The neutralized collagen type-I solution with 3 mg/mL concentration was prepared from 9.1 mg/mL collagen type-I stock solution (Corning, Corning, NY, USA) with a pH of ~3.4 using 1 M NaOH, phosphate-buffered solution (PBS) and deionized water. Then, the primary human fibroblasts (COLO829, ATCC, Manassas, VA, USA) were cultured in a complete media of Eagle’s minimum essential medium (EMEM) (ATCC; Manassas, VA, USA) supplemented with 10% fetal bovine serum (FBS) (Corning; USA) and 1% penicillin-streptomycin (Corning; USA) were encapsulated within the PNCOL with a cell density of 10^6^ cells/mL. After scaffold synthesis and defect area creation, the fibroblast-laden PNCOL solution was injected into the IVD defect area for the experimental group. For the control group, the defect area was left empty. All defect areas were sutured using the modified purse-string suture techniques (MPSS) 1–43. Briefly, two overlapping suture loops were created interconnected and contracted circumferentially for each defect site to provide near watertight sealing. Before starting the organ culture, all samples were submerged in PBS to inspect the sutures for bubbles or PNCOL outflow. 

### 2.4. Ex Vivo IVD Organ Culture

Our established EQUicycler Organ Culture Platform [43] was utilized for culturing the porcine IVDs with and without PNCOL treatment for 14 days within the incubator conditions (37 °C, 5% CO_2_). The EQUicycler Organ Culture Platform can culture ex vivo intervertebral discs and three-dimensional cell-laden musculoskeletal tissue scaffolds under physiologically relevant mechanical and chemical conditions. The organ culture platform can apply various equiaxial mechanical strains up to 15% at different frequencies (up to 2 Hz) without creating damage in cell-laden tissue construct or the ex vivo IVDs for over three weeks [34,48]. Upon damage creation, the IVDs with and without PNCOL were placed inside the organ culture platform chambers. Then, the chambers were filled with Dulbecco’s Modified Eagle Medium (DMEM) media supplemented with 10% fetal bovine serum (FBS), 1% penicillin/streptomycin, 0.5 µg/mL fungizone, and 1:500 primocin. In addition, 1.5% of 5 M NaCl with 0.4 M KCl was added to the cell culture media to prevent swelling. The 10 lbs. static loading was applied to the superior end plate of each IVD for 24 h for 14 days to mimic the in vivo pressure of the disc during the normal posture [49]. The organ culturing was conducted for 14 days, and 50% of the medium was refreshed every three days. After the organ culture period was completed, the IVDs were removed from the chambers for structural, morphological, and biological characterizations. 

### 2.5. Structural and Morphological Characterization

#### 2.5.1. Scanning Electron Microscopy Analysis of Cell-Laden Injectable Tissue Scaffold 

The PNCOL synthesis and collagen fiber structure were assessed using Scanning Electron Microscopy (SEM) (Hitachi, Santa Clara, CA, USA) before scaffold implantation in IVDs. Briefly, before implantation, the cell-encapsulated injectable scaffold was cut and fixed with 4% paraformaldehyde in PBS for 30 min. After fixation, the scaffolds were dehydrated first in sequential ethanol solutions with increasing concentrations from 30% to 100% for 15 min each. Dehydration was then continued by submerging samples into sequential ethanol/hexamethyldisilane (HMDS) solutions from 30% to 100% for 10 min each for image quality enhancement. The samples were then air-dried overnight. The dried samples were then gold-sputter coated and visualized under SEM to observe the morphological and structural changes upon synthesis with fibroblast cells. The fiber diameter calculation of the PNCOL matrix was conducted using SEM images. The SEM images (n = 5) were processed using Fiji/ImageJ (NIH) and over a hundred diameter measurements were taken from each image for calculating the average fiber diameter. 

#### 2.5.2. Histological Analysis of IVDs following the Organ Culturing

The changes in cellularity and collagen morphology were examined using Histological staining. The IVDs were placed in 10% normalized formalin for 48 h. The tissue samples were then processed on a Sakura Tissue TEK VIP 5 tissue processor, dehydrating the samples in 70%, 85%, 95%, and 100% ethanol before being treated in a clearing agent (FISHER XS-05 laboratory-grade Xylene). Following this, the samples were immersed in paraffin (Leica Paraplast Plus) and embedded into a tissue cassette before being cut on a LEICA RM 2235 microtome in 5 mm sections and placed on slides. The slides were incubated in hematoxylin and eosin (H&E) and Masson’s trichrome staining, washed, and viewed under a bright field microscope to observe the cellularity of the IVD after 14 days.

#### 2.5.3. MRI Analysis of IVDs following the Organ Culturing

MRI imaging was carried out with a 3Tesla MRI scanner General Electrics (SIGNA Pioneer, GE Healthcare Systems, Chicago, IL, USA) featuring a Fast Spin-Echo (FSE) pulse sequence imaging protocol. Before image acquisition, IVDs were placed in 10% formalin and stored at 4 °C for 24 h. T2 weighted images of 3 mm slice thickness were acquired following the FSE protocol. Samples were oriented in the coronal plane in a spinal configuration, with the cranial side facing the spinal coil used during the analysis. Imaging was obtained in the transverse planes for each untreated and PNCOL-treated sample after 14 days of organ culture.

### 2.6. Biological Characterization

#### 2.6.1. Cell Viability Analysis

The cell viability was tracked on day 3,7, and 14 using a colorimetric-based CyQUANT XTT cell viability assay (Thermo Fisher Scientific, Waltham, MA, USA). Briefly, the CyQUANT XTT cell viability assay was prepared and kept from light. Triplicates of 100 μL of media extracted from each organ culture chamber were incubated on a 96-well plate mixed with 70 μL of XTT assay reagent. After 8 h of incubation at 37 °C, the OD of the well was determined using a UV kinetic microplate reader at a test wavelength of 450 nm and a reference wavelength of 660 nm. Each plate contained appropriate blank control wells containing media without reagent. Absorbance measurements were calculated based on the vendor’s protocol and extrapolated to the initial time point. At each time point, the data normalized with the initial reading. 

#### 2.6.2. Gene Expression Analysis

After 14 days of organ culturing, the IVDs were dissected for gene expression analysis. The gene expression analysis was conducted using real-time polymerase chain reaction (qRT-PCR). Briefly, on characterization day, IVDs were cut open, and AF tissue was extracted, snapped freeze, and mechanically disrupted. Then, RNA was extracted using TRIzol reagent (Thermo Fisher Scientific, USA). The isolated RNA was reverse transcribed using the Omniscript RT kit (Qiagen, Germantown, MD, USA) per the manufacturer’s instructions. Quantitative real-time PCR was performed using the SYBR Green PCR master mix (Thermo Fisher Scientific, USA) for detecting the expression of hallmark markers of IVD regeneration. The relative gene expression for fold differences between control and PNCOL-treated samples was obtained using the ∆∆Ct method. The ∆∆Ct method used glyceraldehyde-3-phosphate dehydrogenase (GAPDH) as the housekeeping normalizing gene. RT-qPCR was performed in the iCycler iQ detection system (Bio-Rad, Hercules, CA, USA), with thermocycling performed for 35 cycles. The primer sequences were obtained from published literature, as listed in Table 1, and purchased from Integrated DNA Technologies (IDT, Coralville, IA, USA).

### 2.7. Statistical Analysis

Statistical analysis was conducted through R-studio. Statistical analysis was performed using Student’s *t*-test. All values are reported as the mean and ± the standard error of the mean. *p* < 0.05 was statistically different. Symbols on top of each bar profile summarize Tukey’s post hoc analysis of at least three independent experiments. Each study of 4 samples was repeated twice for a total of 8 samples. Four IVDs were assigned as a PNCOL-treated group (n = 4), and another four were assigned as the control group (n = 4) without any nanofiber treatment. At least three technical replicates were produced for each assay performed in this study.

## 3. Results

### 3.1. Confirmation of Annulus Fibrosus Damage Creation

**Figure 3:**
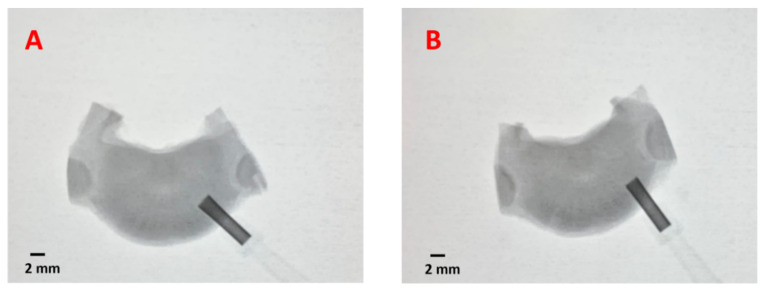
X-ray representative images of the biopsy punch-induced annulus fibrosus defect creation in (**A**) untreated and (**B**) PNCOL-treated IVDs. (n = 4).

X-ray fluoroscopy confirmed the annulus damage creation using a biopsy punch. Figure 3 shows the X-ray images of the biopsy punch position relative to the IVDs during defect creation. 

### 3.2. Structure of Fibrous Cell-Laden PNCOL Scaffold and Cell Viability

**Figure 4:**
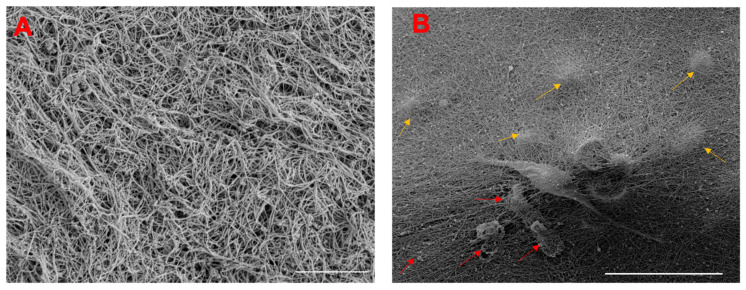
The scanning electron micrographs (**A**) PNCOL without cells, scale bar 10 µm and (**B**) PNCOL with encapsulated cells, scale bar 50 µm. The yellow arrows show the encapsulated cells located under the PNCOL surface, while the red arrows point out the secreted ECM matrix around the cells (n = 4).

Prior to conducting extensive ex vivo IVD organ culturing, we assessed the nanofibrous structure of the PNCOL. Figure 4A shows scanning electron microscope (SEM) images of PNCOL without the cells. The fibrous collagen and PCL nanofibers within the PNCOL provided enough porosity and surface area for accommodating cells within the PNCOL. The average fiber diameter for the PNCOL scaffold was calculated as 0.087 ± 0.01 µm, which provides an excellent topography for improved cell attachment and proliferation [42,53]. Figure 4B also demonstrates a representative image of a cell on the PNCOL surface and the encapsulated cells underneath the PNCOL surface (yellow arrows). In addition, the cells encapsulated within the PNCOL secreted their own extracellular matrix, which covered the porous nanofibrous structure of PNCOL (Figure 4B). The representative secreted matrix around the cells is pointed out in Figure 4B with red arrows. For PNCOL without cells, only the fibrous structure of the PNCOL was observed without secreted ECM matrix. 

### 3.3. Cell Viability within the IVD Organ Culture 

**Figure 5:**
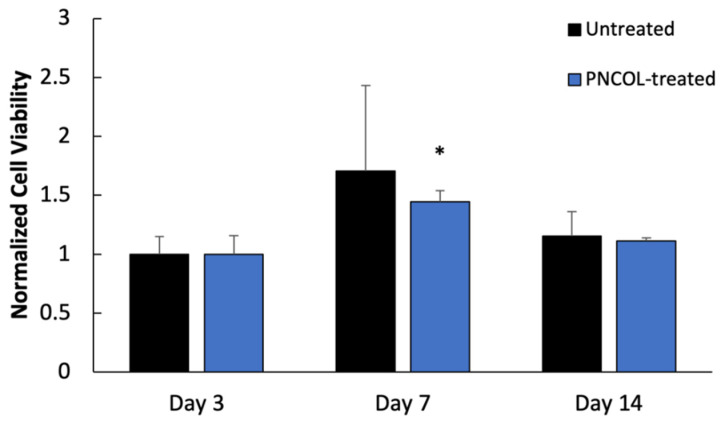
The normalized cell viability was quantified using XTT assay during organ culture for untreated and PNCOL-treated samples (n = 4). * Represents the statistical analysis with Tukey’s posthoc analysis of at least two independent experiments.

For PNCOL-treated and untreated IVDs, the cell viability was measured throughout the organ culture (14 days) period using the colorimetric-XTT assay. Figure 5 demonstrated that the cell viability was preserved throughout the organ culture period for both untreated and PNCOL-treated IVDs. For PNCOL-treated IVDs, the cell viability was slightly increased on day 7 with a statistically significant level (*p* < 0.05) but there was no statistical difference between day 7 and day 14. For untreated IVDs, no statistical changes in cell viability were observed based on XTT data. 

### 3.4. Optical and Histological-Based AF Tissue Regeneration Analysis

**Figure 6:**
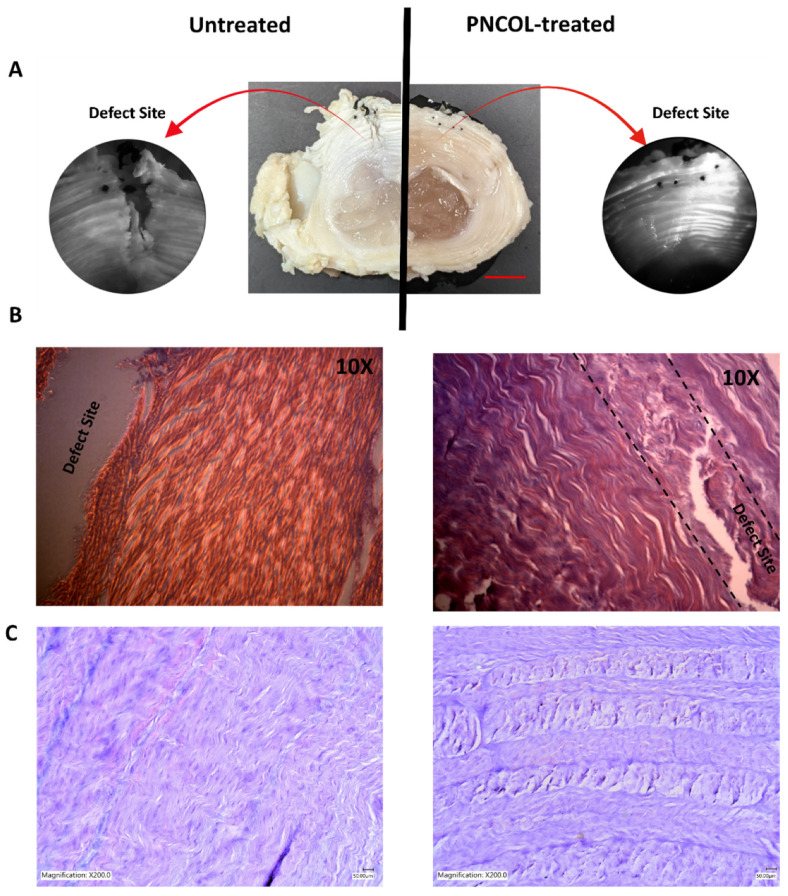
(**A**) The optical images of untreated and PNCOL-treated IVDs, along with zoom-in optical images from the defect sites. Scale bar is 1 cm. (**B**) Representative histological images with defect sites taken with 10× magnification and (**C**) Representative histological images with defect sites taken with 200× magnification from untreated and PNCOL-treated IVDs histological slides (n = 4).

Immediately after the organ culture study was completed, the vertebral bony section and the endplate of the IVDs were removed to observe the morphological differences between the untreated and PNCOL-treated annulus fibrosus section. The gross optical images demonstrated significant morphological differences between PNCOL-treated AF and untreated counterparts (Figure 6A). The PNCOL-treated AF group demonstrated a fully repaired AF structure with the reintegration of broken AF rings at the defect site. In contrast, the defect area within the control group did not heal, and the puncher defect was still visible (Figure 6A) upon 14-day organ culturing. To further understand the effect of PNCOL treatment on AF tissue healing, the histological analysis through zoom-in histological slides pictures with 10x magnification was conducted on untreated and PNCOL-treated IVDs following optical imaging. For untreated IVDs, the histology images (Figure 6B) showed a significant lack of tissue regeneration, specifically with the visible defect through the AF.

On the other hand, the defect site almost completely healed for PNCOL-treated AF tissues. The defect site for PNCOL-treated AF was shown between two dashed lines in the 10X histological images (Figure 6B) because there was a minimal remnant of the defect site, unlike counterparts in untreated AFs. The fibrous structure of cell-laden-PNCOL is attached completely and integrated well with the native tissue at the defect site without a gap at the interface. The newly generated matrix at the defect area started to remodel itself like native lamella fibrous AF tissue around it following 14 days of organ culturing (Figure 6B,C). 

### 3.5. MRI Analysis 

**Figure 7:**
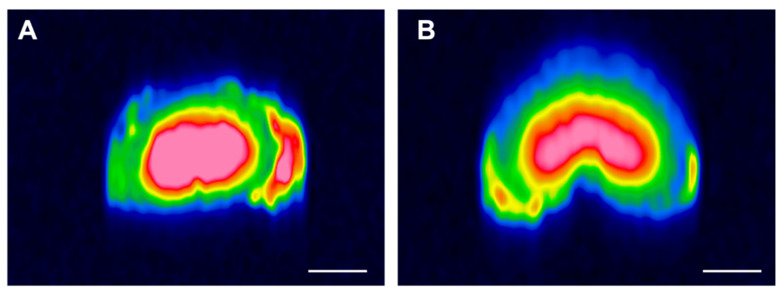
MR images were obtained post-experiment. (**A**) Transverse plane image of untreated IVD, (**B**) Transverse plane image of PNCOL-treated IVD. Images represent a 3 mm-thickness slice. Scale bar is 1 cm (n = 4).

MRI-Structural imaging was achieved by T2 weighted images for all IVDs. Among all 3 mm-thickness slices, a representative slice for each group was selected (Figure 7). MRI data displayed a significant difference between the untreated and PNCOL-treated IVDs after 14 days of organ culture. The PNCOL-treated IVDs showed a clear, distinct, and continuous border between the NP and AF (Figure 7B) in the transverse plane. Additionally, the PNCOL-treated samples demonstrated that the fibrous ring in the periphery of the IVD was healed along with the defect site with no sign of herniations. In contrast, the untreated IVDs showed herniations at the defect site, with the NP protruding beyond the proximal layers of the AF (Figure 7A). 

### 3.6. Molecular Analysis

**Figure 8:**
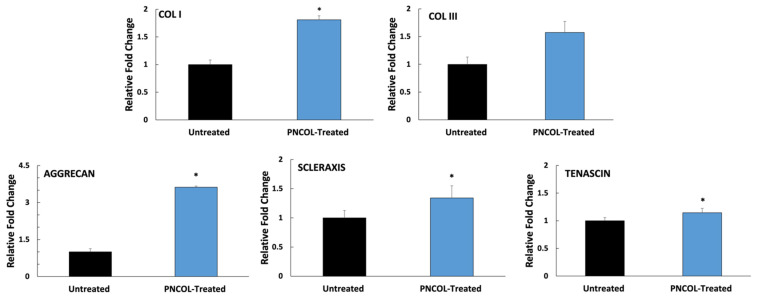
The relative fold changes of AF extracellular markers of untreated (control) and PNCOL-treated IVDs following 14 days organ culture. All data were expressed as the mean, and standard deviation. * indicates the statistical differences between groups with *p* < 0.05, n = 4, and at least three technical replicates were used for gene expression analysis.

To understand the effect of PNCOL treatment on AF tissue at the molecular level, a comprehensive gene expression analysis was conducted on PNCOL-treated and untreated IVDs in all replicated experiments. At least three samples were used in each experiment. Figure 8 shows the relative gene expression fold change of important AF ECM markers (Collagen I, III, Aggrecan, Scleraxis, Tenascin) [34,54,55,56,57] between untreated and PNCOL-treated IVDs following 14 days organ culturing. For PNCOL-treated AF, the gene expression of Collagen I (COL I), Aggrecan, Scleraxis, and Tenascin upregulated was statistically significant compared to untreated AF. Yet, collagen III (COL III) gene expression showed no significant difference in the PNCOL-treated and untreated AF. In particular, the aggrecan gene expression demonstrated a higher upregulation with a 3.6 ± 0.09-fold change in the PNCOL-treated IVDs, demonstrating enhanced AF regeneration. 

**Figure 9:**
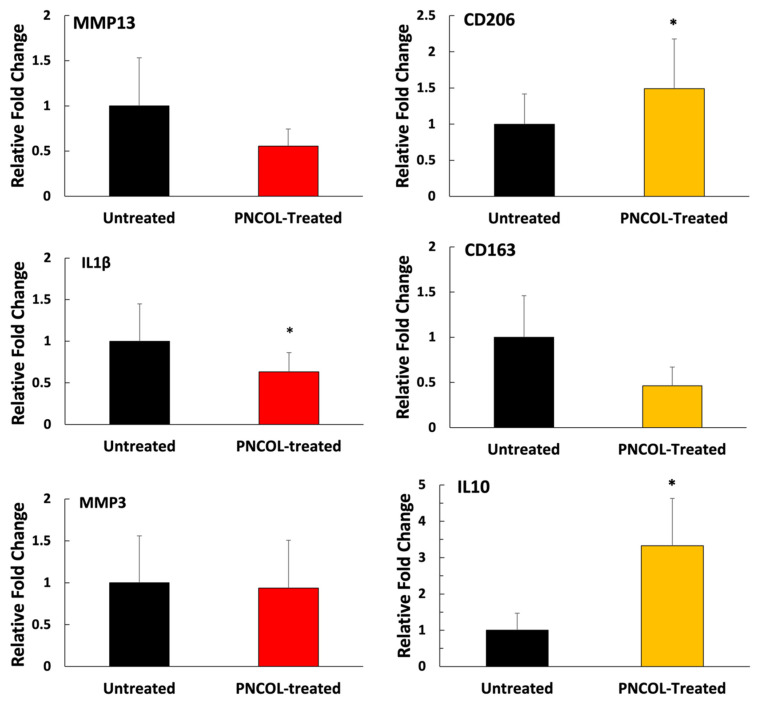
The relative fold changes in MMP13, pro- and anti-inflammatory markers within the untreated and PNCOL-treated IVDs following 14 days organ culture. All data were expressed as the mean, and standard deviation. * Indicates the statistical differences between groups with *p* < 0.05, n = 4, and at least three technical replicates were used for gene expression analysis.

Further, the effect of PNCOL treatment on extracellular matrix-degrading enzyme MMP13 gene expressions and pro- and anti-inflammatory gene expressions were analyzed. The gene expression analysis of extracellular matrix-degrading enzyme MMP13 demonstrated that PNCOL treatment of defect IVD did not trigger MMP13 upregulation (Figure 9). There was no statistical difference in MMP13 expression of PNCOL-treated and untreated AFs following 14 days of organ culture. For anti-inflammatory markers, PNCOL treatment of defect AF statistically upregulated the expression of CD206 and IL10 with 1.5 ± 0.68 and 3.3 ± 1.29-fold change, respectively. However, there were no statistically significant changes in expressions of CD163 between PNCOL-treated and untreated IVDs. In addition, the PNCOL- treated IVDs demonstrated changes in expressions of pro-inflammatory markers, IL1β and MMP3, compared to untreated IVDs. The gene expression analysis showed that both IL1β and MMP3 expressions were downregulated in the PNCOL-treated IVDs. Yet only the IL1β expression in PNCOL-treated IVDs demonstrated a statistical significance decrease (*p* < 0.05) compared to the untreated IVDs.

## 4. Discussion

Intervertebral disc degeneration plays a crucial role in low back pain (LBP), which causes an immense socioeconomic burden on society [58]. Overwhelming evidence suggests that current surgical procedures inadequately solve the LBP problem because of a failure to address IVD degeneration. Therefore, there is a great need for an AF closure therapy that actively promotes disc regeneration through revitalizing the AF tissue [28,58]. Our prior in vitro studies [34,42,53] demonstrated that cell-laden PCL nanofiber-incorporated collagen (PNCOL) scaffold promotes musculoskeletal tissue regeneration, including annulus fibrosus, and accommodates encapsulated cell population through its nanofibrous yet injectable structure. Yet, it was unclear whether promising in vitro results obtained in PNCOL-induced AF tissue regeneration could be translated into ex vivo tissue regeneration in organ culture conditions. To this end, in this study, we investigated whether PNCOL scaffolds seeded with fibroblast cells could treat defected porcine AF tissue under an ex vivo IVD organ culture environment using our well-established IVD organ culture platform [48]. 

Following X-ray-guided defect creation, the cell-laden PNCOL scaffolds were introduced to the defect area, while the untreated defect area served as a control. The nanofibrous structure of PNCOL scaffolds (Figure 4A) promotes cell attachment and lamellipodia formation (Figure 4B). The SEM images of cell-laden PNCOL demonstrated the encapsulated cells on the PNCOL surface and within the PNCOL. Unlike only the PNCOL SEM image (Figure 4A), the cell-laden PNCOL SEM image (Figure 4B) shows the extracellular material secreted by encapsulated cells already proved in our prior studies [41]. 

To further study the role of cell-laden PNCOL in AF regeneration following 14-day organ culturing, the IVD was sectioned in the transverse plane, and the endplate was removed for both untreated and PNCOL-treated IVDs. The gross optical images of the defect site (Figure 6A) demonstrated that in the absence of treatment, a biopsy punch induced a degenerative fissure that a native IVD could not repair without intervention. On the other hand, the same fissure brimmed with newly regenerated tissue when the cell-laden PNCOL scaffold was introduced to the defect site. The gross optical images of the defect site (Figure 6A) demonstrated that cell-laden PNCOL regenerated the AF tissue, which was further proved with histological analysis of AFs defect sites. The histological data showed that for AF treated with a cell-laden PNCOL scaffold, not only was the tissue at the defect site fully regenerated, PNCOL scaffold uniquely promoted the lamellar tissue formation (Figure 6B,C) that mimics and assimilates into native AF fibers and rings. The regenerated AF tissue in the cell-laden PNCOL treatment group has thicker, denser, and more organized fibers aligned with lamellae rings compared to counterparts in the untreated group (Figure 6C).

Further, the AF tissue regenerated by cell-laden PNCOL scaffolds demonstrates alignment with the native AF tissue (Figure 6B,C). Yet, the collagen within the untreated AF tissue was disorganized, thinner, and fraying, indicating degeneration around the defect area [59,60]. The magnetic resonance images (MRI) of untreated and cell-laden PNCOL-treated IVDs further strengthen the histological findings. The MRI data demonstrated AF healing at the defect site with well-defined lamella rings (Figure 7). On the other hand, the untreated IVDs did not have a clear AF border and demonstrated a distorted NP section. In all, histological analysis and MRI data support that cell-laden PNCOL scaffold promoted new AF tissue regeneration at the defect area with organized and robust ECM formation. A gene expression analysis was conducted to understand the cellular responses and molecular effects of cell-laden PNCOL treatment at the AF defect. The gene expression results (Figure 8 and Figure 9) revealed that PNCOL treatment in AF defect significantly increased important ECM markers that indicate wound healing (Tenascin and Scleraxis) [1,61] and structural integrity (Aggrecan and Collagen-I). The increased gene expression of tenascin in the PNCOL-treated IVDs suggests enhanced wound-healing abilities as tenascin is expressed during tissue healing [62] and has roles in cell signaling and promoting cell migration and proliferation [63,64]. Tenascin’s significant upregulation demonstrates that the PNCOL-treated IVDs were able to initiate a healthy cellular response more effectively and are better able to promote tissue regeneration by guiding cellular proliferation and increasing ECM components compared to untreated IVDs. Similarly, the upregulation of scleraxis in PNCOL-treated IVDs demonstrated that the PNCOL treatment with fibroblast cells promoted AF ECM regeneration. Scleraxis is a critical transcription factor during tissue regeneration and has been shown to stimulate proper healing processes in fibrous tissues that require specific fiber organization and production [65,66].

In addition to wound healing, the nutrition of new tissue is vitally important in any effort to revitalize the disc. Proteoglycans such as aggrecan play a crucial role in tissue nutrition. As aggrecan accumulates, it creates an osmotic gradient that pulls in nutrients from the vertebral endplate, thereby drawing pressure to counteract compressive loads and drawing in nutrients from the vertebral endplates above and below the IVD [67]. Additionally, aggrecan can form polymers with other glycosaminoglycans and gives IVDs the capacity to resist high compressive loads without collapsing or damaging the tissue [68]. Because of these vital functions, reduced aggrecan within the IVD is a principal factor in the intervertebral disc degeneration [69,70]. The significant upregulation in aggrecan gene expression (Figure 8) upon PNCOL treatment suggests that PNCOL treatment of damaged IVD promoted essential AF ECM protein. Gene expression analysis also demonstrated that PNCOL-treated AF tissues statistically significantly upregulated the collagen type-I expression, a key ECM marker commonly associated with mechanical strength in the AF. The increased expression of collagen type-I suggests increased structural stability for the outer annulus fibrosus [71,72]. Clinically, this has been shown to translate to a reduction in the rate of herniation and less pain for post-operative patients [73,74]. The PNCOL incorporation into the defect not only increased the AF matrix regeneration but also upregulated the anti-inflammatory markers IL10 and CD206 expressions and significantly (*p* < 0.05) downregulated the potent pro-inflammatory marker of IL1β (Figure 9), which can be served as an immunomodulatory biomaterial. The overall downregulation of pro-inflammatory markers and the decreased expression of IL1β agree with the numerous studies suggesting that anti-IL1β treatment of degenerated IVD leads to tissue regeneration [7,8]. The PNCOL-induced anti-inflammatory and ECM gene upregulation helped AF tissue regeneration at the injury site, further confirmed in MRI (Figure 7) and histology analysis (Figure 6B,C).

This study provides a great avenue for investigating cell-laden injectable PNCOL scaffold for AF tissue regeneration, but it also has some limitations. For instance, the amount of secreted de novo collagen within the scaffolds was not assessed, which could further prove the superior tissue regeneration capacity of cell-laden PNCOL matrix. The current study could also benefit from protein-level analysis, such as Western blotting for synthesizing key ECM and pro- and anti-inflammatory markers. For future work, this study can be extended further by incorporating growth factors such as fibroblast growth factor (FGF) and anti-inflammatory cytokines such as IL-4 [75] within the PNCOL scaffolds to enhance AF tissue regeneration around the defect area during the IVD organ culturing. Incorporating the PNCOL matrix without cells into the defective AF tissue can also be an interesting study to understand the solo effect of the PNCOL matrix on AF tissue regeneration in organ culture. In addition, the longitudinal study in AF regeneration in organ culture can be beneficial for understanding how PNCOL-induced AF regeneration progresses over time and how the expression and synthesis of key AF ECM markers and pro-and anti-inflammatory markers change with different time points.

## 5. Conclusions

In conclusion, the fibroblast-laden PNCOL scaffold can restore natural AF tissue at the defect site following 14 days of ex vivo porcine IVD culturing. The PNCOL-treated AF defects demonstrated normal coloration, higher collage matrix organization, and ECM anabolic states, all reflective of IVD regeneration confirmed using structural, molecular, and cellular analysis. The cell-PNCOL scaffold presents a viable non-invasive therapeutic option for AF regeneration which was assessed using clinically relevant big animal (porcine) IVD organ culturing.

## Figures and Tables

**Table 1:** Forward and reverse primers for real-time PCR.

Gene	Forward Primer	Reverse Primer	Ref.
*Scleraxis*	AGCAACCAGAGAAAGTTGAGCA	CTGCCTGTCTGTCCATTGGC	[50]
*Col I*	TCCCTGGTGCTGTTGGTG	TACCAGGAGCGCCGTTG	[50]
*Col III*	CGACTTCTCTCTAGCCGAGC	CCCCAGTGTGTTTAGTGCAAC	[51]
*Tenascin*	AGGTTCCTGGAGACGATGGA	TCTGGGGTGGCATCTGAAAC	[52]
*Aggrecan*	CAGGTGAAGACTTTGTGGACATC	GTGAGTAGCGGGAGGAGCCC	[50]
*MMP13*	CATGAGTTTGGCCATTCCTT	GTGGCTTTTGCCAGTGTAGG	[50]
*IL-10*	GCCTTCGGCCCAGTGAA	AGAGACCCGGTCAGCAACAA	[50]
*CD163*	TCTGTTGGCCATTTTCGTCG	TGGTGGACTAAGTTCTCTCCTCTTGA	[50]
*IL1* *β*	CCAGTACGAATCTCGGACCACC	TTAGGAAGACACAAATTGCA	[50]
*MMP3*	TCCTGATGTTGGTTACTTCAGCAC	TTGACAATCCTGTAAGTGAGGTCATT	[50]
*CD206*	CTACAAGGGATCGGGTTTATGGA	TTGGCATTGCCTAGTAGCGTA	[50]
*GAPDH*	GTTTGTGATGGGCGTGAACC	AGCTTGACGAAGTGGTCGTT	[50]

## Data Availability

The datasets generated during the current study are not publicly available due to the pending patent application but are available from the corresponding author upon reasonable request.
